# The Use of Antiseptics for Sterilizing Cavities before Filling

**Published:** 1892-03

**Authors:** H. A. Smith

**Affiliations:** Cincinnati, O.


### The Use of Antiseptics for Sterilizing Cavities before Filling.

BY H. A. SMITH, D.D.S., CINCINNATI, O.

Read before the Ohio State Dental Society, held at Columbus, December, 1891.

My purpose in this paper is to consider briefly the use of antiseptics in sterilizing carious dentine in deep seated cavities.

It is now regarded as good practice to leave in the bottom of cavities a layer of carious dentine if by its removal the pulp should become exposed. In such cases the continued health of the pulp depends upon the thorough sterilization of this layer. Upon this point, Prof. Black says: "Where we cover in a little bit of softened dentine over a pulp nearly exposed, we may cover in the anærobic microbes. In a short time they may produce products that will destroy the pulp, or they pass through and penetrate into the pulp and infect it. This action is brought about rapidly and the poisonous matter escapes towards the pulp. If we have covered these microbes in with the filling we have sealed up the elements for destroying that pulp. So here we need an antiseptic."

In considering this subject, naturally the first inquiry would be, what chemical or physical changes have taken place in this layer? Dental caries suggests decalcification. The degree to which decalcification has taken place depends upon which portion,—the superficial, the middle, or the deeper seated portion—of softened dentine is under examination. Prof. Miller in his recent work gives the result of his investigations upon the whole mass of softened dentine in the cavity, to ascertain the comparative loss of the organic and inorganic constituents. After giving his methods and the result of analysis, he says, "In plain words the carious dentine had suffered an almost complete decalcification—only one-thirteenth of the original amount of lime salts being present. The organic matter had suffered the comparative small loss of two-fifths of its original amount. This loss is no doubt attributable for the most part to the direct action of micro-organisms upon the more completely decalcified portions of the carious dentine." Continuing, he says, "The results of these experiments show that the organic matter yields last to the destroying agents." It will be seen, then, the layer of carious dentine which we propose to sterilize, is, more or less, composed of the organic matter which was originally the basis substance of the dentine. Therefore it is albuminous, and if tested, should give an acid reaction.

In selecting an antiseptic for the purpose indicated, the length of time the antiseptic may be permitted to remain in the cavity before introducing the filling, should be considered. If only for a few minutes, or during the excavation of the cavity, the antiseptic should be one that will not coagulate albumen and one that is quickly diffusible. In some of the oily antiseptics we have those that meet these conditions, preferably the oil of cassia, oil of cloves, oil of turpentine, and eucalyptol. The diffusibility of these oils is conclusively shown by the experiments of Prof. Harlan. His conclusions are also fully borne out by clinical experience. In cases requiring immediate treatment, after thoroughly drying I usually apply oil of cassia and oil of cloves, equal parts. If the carious layer is of considerable thickness would apply oil of cassia alone. The latter being somewhat irritating, the addition of oil of cloves will, in a degree, modify this action, if liable to come in contact with pulp tissue. Myrtol has been highly recommended by Prof. Harlan because of its being a very pleasant, non-irritating and highly potent antiseptic. Those who believe the ideal antiseptic should be soluble in water, may use carbolic acid, bichloride of mercury, terchloride of iodine, or the latest addition to this class of agents, lysol, a perfectly soluble antiseptic of the cresol group. Of the above, carbolic acid is most frequently used, and I have no doubt that in many cases if permitted to remain sealed in the cavity several hours, from 5 to 10, will effectually sterilize carious dentine. To overcome in part the coagulating properties of carbolic acid, I have been in the habit of using a mixture of carbolic acid one part, oil of cassia two parts, oil of cloves three parts. In cases when a permanent filling must follow treatment at the same sitting I find the above modification of Prof. Black's 1, 2, 3 mixture very satisfactory.

I have already referred to the need of dryness in the antiseptic treatment of this layer of carious dentine. This cannot be too strongly insisted upon. If the layer, especially the upper and more albuminous portion is saturated with moisture, the diffusion of any of the antiseptics, would be greatly retarded; besides, if an antiseptic readily soluble is used, its effective strength may be reduced to a degree which renders it inert. In the layer nearest the pulp, where we may suppose caries is still active, the normal tubular structure of dentine is more nearly maintained. These minute tubes are a physical barrier to the diffusion of an antiseptic, whether in solution, an oil, or an emulsion. And if the moisture which is natural to the protoplasmic contents of the tubules, or to the micro-organisms in them is not removed in greater part, the diffusion of the antiseptic is still further interfered with. How may we obtain this dryness? By bathing the layer with an agent which has an affinity for water, as alcohol, and evaporating it with warmed or heated air.

The methods practiced to obtund sensitive dentine by dehydration are usually efficient, and if carefully followed out, by the time the cavity is prepared, we will have desiccated the layer of carious dentine in the bottom. The use of alcohol for this purpose may be objected to because of its coagulating effects upon albumen. This property, however, would be very slightly exhibited, because of the rapidity with which it would be evaporated by the hot air blast.

It may be in order, in this connection, to refer to a class of cases in which caries recurs after the tooth is filled, because of some defect at the margin of the cavity. Caries advances along the wall of the cavity until the bottom is reached. Here we find a softened portion of dentine extending partially under the filling. Often, because of the difficulty in reaching this diseased portion, the filling is removed and the whole operation done over. This in some instances may be avoided provided the layer of carious dentine beyond the reach of the excavator can be sterilized. To accomplish this, the whole of the softened dentine must be thoroughly dried, the antiseptic applied and the opening carefully sealed for a day or two, after which permanent repair of the filling may be made.

It may be said, lack of thoroughness in the removal of carious dentine, begets carelessness in our methods of practice, and yet, if partially decalcified dentine on the walls of the cavity well away from the margins, may be made fixed matter, why may it not be left?

In the class of cases above described, the difficulties in sterilization increase in proportion with the increase in thickness of the layer of softened dentine under treatment. For this reason, and because of a lack of knowledge of the relative potency of the various antiseptics we use, it would be well, perhaps, to restrict their application to the sterilization of layers of carious dentine left in the bottom of cavities for pulp protection.

